# Cardiovascular Autonomic Control in Normotensive Patients with Autosomal Dominant Polycystic Kidney Disease

**DOI:** 10.34067/KID.0000000958

**Published:** 2025-08-28

**Authors:** Daniel Ribeiro Rocha, Ana Carolina Anauate, Milene Subtil Ormanji, Cássia Toledo Bergamaschi, Ruy Ribeiro Campos, Bruno Moreira Silva, Ita Pfeferman Heilberg

**Affiliations:** 1 Nephrology Division, Universidade Federal de São Paulo, São Paulo, Brazil; 2 Department of Physiology, Universidade Federal de São Paulo, São Paulo, Brazil

**Keywords:** ADPKD, angiotensin, BP, cardiovascular, clinical hypertension, genetic renal disease, hypertension, polycystic kidney disease, renin-angiotensin system

## Abstract

**Key Points:**

- Systemic arterial hypertension is a frequent early onset manifestation in autosomal dominant polycystic kidney disease (ADPKD) contributing to progression.
- Assessment of cardiovascular autonomic function in young patients with ADPKD before hypertension or renal decline revealed no abnormalities.
- Findings showed no autonomic dysfunction, but high urinary angiotensinogen suggests early intrarenal renin angiotensin-aldosterone system activation in normotensive patients with ADPKD.

**Background:**

Autosomal dominant polycystic kidney disease (ADPKD) is the most common monogenic kidney disease, leading to progressive renal function loss. Systemic arterial hypertension is a frequent early-onset extrarenal manifestation with an incompletely understood pathogenesis. Therefore, this study investigated cardiovascular autonomic control at rest and during physiological sympathetic stimulation, along with humoral and urinary molecules involved in BP regulation, in young patients with ADPKD before hypertension and renal dysfunction onset.

**Methods:**

Eighteen normotensive patients with ADPKD (11 female/7 male, 27.7±6.4 years) and 19 age- and sex-matched healthy controls (9 female/10 male, 25.7±3.8 years) participated in this study. Based on Mayo Clinic imaging criteria, patients with ADPKD were classified as rapid or slow progressors. Heart rate variability, BP variability, and spontaneous baroreflex sensitivity (BRS) were assessed at rest, with BRS further evaluated during the Valsalva maneuver. Cardiovascular reactivity to sympathoexcitation was examined using the cold pressor test, Stroop test, and static handgrip exercise. Neuropeptide Y, angiotensinogen (AGT), and inflammatory and endothelial function markers were measured in blood, whereas monocyte chemoattractant protein-1, AGT, and albumin were analyzed in urine.

**Results:**

Heart rate variability, BP variability, BRS, and cardiovascular reactivity did not differ between patients and controls or between rapid and slow progressors. Serum markers and urinary monocyte chemoattractant protein-1 were also not different between groups. However, urinary AGT and albumin levels were significantly higher in patients.

**Conclusions:**

These findings suggest that cardiovascular autonomic dysregulation, systemic inflammation, and endothelial dysfunction are absent in early-stage ADPKD, whereas intrarenal renin-angiotensin-aldosterone system is overactivated and potentially plays a key role in triggering hypertension.

## Visual Abstract

**Figure:**
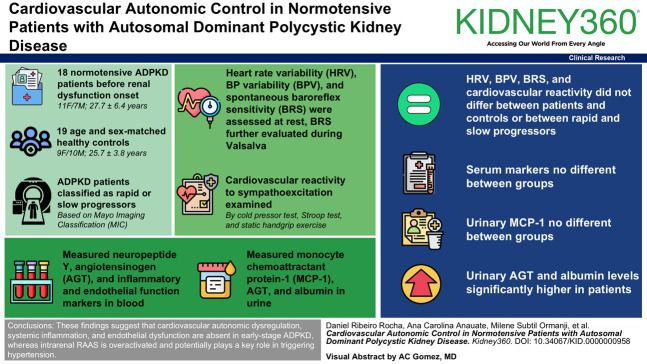

## Introduction

Autosomal dominant polycystic kidney disease (ADPKD) is characterized by the continuous formation of kidney cysts and progressive loss of renal function. It is the most prevalent monogenic kidney disease, affecting approximately 2–6.8 per 10,000 individuals,^1–4^ and accounts for about 10% of patients undergoing KRT.^5^ Cardiovascular disease is a major cause of morbidity and mortality in patients with ADPKD,^6,7^ with systemic arterial hypertension being the most common extrarenal noncystic manifestation involving the cardiovascular system.^8,9^ Hypertension has an early onset, affecting 50%–70% of patients at a young age, even before kidney function declines.^10,11^

The pathogenesis of hypertension in ADPKD is multifactorial and not fully understood. It is hypothesized to involve both systemic and intrarenal activation of renin-angiotensin-aldosterone system (RAAS) as well as extrarenal abnormalities such as systemic inflammation, endothelial dysfunction, and autonomic dysregulation.^12–18^ Studies in animal models suggest that autonomic dysregulation of the heart and systemic resistance vessels occurs early in kidney diseases, probably resulting from abnormalities in both neural and humoral pathways associated with the development of hypertension in the diseased kidneys.^19–22^ Potential mechanisms include increased afferent renal signaling,^23^ elevated circulating angiotensin II acting on carotid bodies,^24^ and circumventricular organs,^25^ as well as systemic inflammation^26^ and vascular dysfunction^27^ impairing central autonomic neurons and autonomic ganglia function.

However, the hypothesis considering cardiovascular autonomic dysregulation as a trigger of BP elevation in ADPKD remains uncertain in humans because most studies in the area did not assess patients at an early phase of the disease.^28–31^ The autonomic nervous system regulates the heart in addition to peripheral vessels, and some of its alterations are only revealed prematurely during sympathoexcitatory maneuvers,^32–35^ a potential autonomic dysfunction in an early phase of ADPKD natural history cannot be discarded. Moreover, the influence of total kidney volume (TKV), a contemporary marker of disease progression,^36,37^ on the cardiovascular autonomic regulation in normotensive patients with ADPKD remains unexplored.

Therefore, this study aimed to investigate cardiac and vascular autonomic control at rest and during physiologic sympathetic stimulation in a cohort of young patients with ADPKD, before the development of hypertension and renal dysfunction.

## Methods

### Study Population

Nineteen adult patients (age 18–40 years) diagnosed with ADPKD based on the Pei ultrasonographic criteria and a positive family history^38^ were included. All patients were followed at the ADPKD outpatient clinic of our university. They were normotensive and had an eGFR ≥60 ml/min per 1.73 m^2^ and a body mass index (BMI) <30 kg/m^2^. We excluded patients with a history of cardiovascular disease, diabetes mellitus, asthma, smoking, alcohol consumption, or psychiatric disorders. One patient was later excluded because of electrocardiogram abnormalities during the experimental visit, leaving 18 patients who completed the study. A control group of 19 healthy individuals, matched for sex and age, were included after confirming normal renal function and ruling out systemic arterial hypertension, obesity, or other diseases. The study was approved by the Local Clinical Research Ethics Committee (Approval No. 3.659.766 [0802/2019]). Written consent was obtained from all participants.

### Autonomic Assessment

#### Resting State

It is the measurement of heart rate variability (HRV), BP variability (BPV), and spontaneous arterial baroreflex sensitivity (BRS) during 5 minutes of spontaneously breathing at rest. Electrocardiogram and beat-by-beat BP were continuously recorded for 5 minutes during seated rest with spontaneous breathing, following a monitoring protocol as previously described by our group.^39^ Time-domain and frequency-domain HRV and BPV analysis are demonstrated, and some indexes have significant autonomic support, as demonstrated by physiologic, pharmacologic, surgical, and other manipulations in animals and humans.^40^ SD and the root mean square of successive differences between RR intervals (refers to the time between two consecutive R waves on an electrocardiogram) and high frequency of HRV are largely determined by the vagal modulation of sinoatrial depolarization.^41^ Low frequency of HRV is influenced by both vagal and sympathetic modulation of sinoatrial depolarization;^42^ SD and low frequency of BPV are dependent on sympathetic vasomotor drive.^43,44^ BRS was analyzed using both spectral^45^ and sequence methods^46,47^ *via* CardioSeries software (v2.4, https://cardioseries.software.informer.com/).

#### Valsalva Maneuver

It is the measurement of BRS during Valsalva maneuver (VM)–induced BP changes. Individuals blew into the mouthpiece of an aneroid manometer at 40 mm Hg for 15 seconds while BP and heart rate (HR) were continuously recorded. HR and BP variations during and after the VM were categorized into four phases.^48,49^ Baseline systolic BP (SBP) was measured over 60 seconds before the maneuver. Pressure recovery time (PRT) was defined as the time from the nadir of phase 3 until SBP returned to baseline. Adrenergic BRS (BRSa) was calculated as the SBP decrease during phase 3 divided by PRT.^50^ Vagal BRS (BRSv) was assessed using the slope of the regression between RR intervals and SBP in phase 2^51^ and the Valsalva ratio, the ratio of the highest HR in phase 2 to the lowest HR in phase 4.^52^ The product of BRSv and BRSa was calculated as BRSg, a global measure of baroreflex function.^50^

#### Sympathoexcitatory Maneuvers

It is the measurement of cardiovascular reactivity to mental stress, the cold pressor test (CPT), and static handgrip exercise. Cardiovascular parameters were recorded for 2 minutes before and during three sympathoexcitatory maneuvers. The test order was randomized for each participant, with a minimum interval of 10 minutes between tests. Cardiovascular reactivity was defined as the highest change in 10-second mean values of SBP, diastolic BP (DBP), and HR (*i.e*., delta Δ) during each maneuver compared with baseline values. This reactivity is largely mediated the sympathetic branch of the autonomic nervous system.^53–55^ Most importantly, it can predict hypertension development in otherwise normotensive healthy individuals.^32–35^

### Stroop Test

Participants viewed color names displayed in different ink colors (incongruent task) on a monitor, with varying background colors and audio of spoken color names. The speed of presentation gradually increased to induce mental stress. Participants were asked to say the ink color of each word, ignoring the written word, background, and spoken color. Mental stress intensity was then assessed using a visual analog scale from 0 to 10.

### Handgrip

Handgrip strength was measured with the participant seated, elbow at 90°, forearm neutral, and wrist at 0°–30° extension. First, maximum grip strength was tested by having the participant exert maximum force with the dominant hand for 3 seconds, repeated until two values within 2 kg were obtained. Then, participants maintained 30% of their maximum force for 2 minutes. Perceived effort was assessed using the Borg CR-10 scale at 1 and 2 minutes.

### CPT

Participants were asked to immerse their dominant hand, palm and back, in a container (thermal box) with ice water at a temperature of 0°C–3°C for 2 minutes. Water temperature was monitored using a thermometer. A visual analog scale ranging from 0 to 10 was used to assess pain intensity at every 30 seconds during the CPT.

### Blood and Urine Measurements

Routine tests included serum creatinine, total cholesterol and its fractions, sodium, and potassium. Serum creatinine was used to calculate the eGFR using the CKD Epidemiology Collaboration 2021 equation.^56^ Blood samples were analyzed for IL-6, TNF-*α*, neuropeptide Y (NPY), asymmetric dimethylarginine (ADMA), and angiotensinogen (AGT; serum AGT [sAGT]). Urine samples were analyzed for AGT (uAGT) and monocyte chemoattractant protein-1 (MCP-1). ELISA kits were used to measure serum IL-6, TNF-*α*, NPY, and urinary MCP-1 (R&D Systems); plasma ADMA (Immundiagnostik, Germany); and sAGT and uAGT (IBL, Japan), following the manufacturer's instructions. Urinary creatinine levels were measured to correct AGT (uAGT/uCr), albuminuria (uAlb/uCr), and MCP-1 (uMCP-1/uCr) values.

### TKV

Data from nonenhanced abdominal magnetic resonance imaging were used to determine TKV adjusted for height (HtTKV) through ellipsoid equation and Mayo Clinic Imaging Classification (MIC), as proposed by Irazabal *et al*.^37^ These data were retrieved from the medical records of patients with ADPKD.

### Statistical Methods

Categorical variables are presented as absolute and relative frequencies, whereas numerical variables are expressed as mean±SD, following verification of normality using the Shapiro–Wilk test. If normality was not met, variables were log-transformed (natural logarithm) to enable parametric testing. In such cases, data were reported as median (interquartile range). The chi-square test was used to assess associations between categorical variables. Mean comparisons between two groups were conducted using the Student *t* test for independent samples. Within-group comparisons were performed using two-way mixed ANOVA with repeated measures, provided normality was confirmed. Pearson correlation was applied to examine relationships between continuous variables. A significance level of 5% was adopted for all statistical tests. Data analyses were conducted using the Jamovi statistical package (version 2.3, https://www.jamovi.org).

## Results

### Characteristics of Participants

No significant differences were observed between groups in terms of sex distribution, age, BMI, or eGFR (Table 1). The average time since the ADPKD diagnosis was 8.7±4.9 years. Resting hemodynamic and biochemical parameters also did not differ between groups.

**Table 1:** Baseline clinical, hemodynamic, and laboratory characteristics of the participants

Characteristics	ADPKD (*n*=18)	Controls (*n*=19)	*P* Value
Female, *No.* (%)	11 (61.1)	9 (47.4)	0.40
Age, yr	27.7±6.4	25.7±3.8	0.24
BMI, kg/m^2^	23.5±4.1	23.3±1.4	0.90
Time since ADPKD diagnosis, yr	8.7±4.9	—	—
**Hemodynamic variables**			
HR, beats/min	73.8±10.4	74.9±8.7	0.75
SBP, mm Hg	128.1±16.5	126.5±12.9	0.75
DBP, mm Hg	82.6±10.9	80.6±9.9	0.57
**Laboratory tests**			
Creatinine, mg/dl	0.91±0.17	0.93±0.11	0.72
eGFR, ml/min per 1.73 m^2^	101.5±17.6	102.1±11.4	0.91
Cholesterol, mg/dl	166.7±31.2	163.4±32.2	0.77
LDL, mg/dl	98.2±22.3	90.9±32.3	0.48
HDL, mg/dl	45.5±11	54.5±13.8	0.06
Na, mEq/L	138.3±2.5	138.4±2.0	0.92
K, mEq/L	4.2±0.4	4.2±0.2	0.85

Values are means±SD. Groups were contrasted *via* the independent samples Student *t* test or chi-square. ADPKD, autosomal dominant polycystic kidney disease; BMI, body mass index; DBP, diastolic BP; HR, heart rate; K, potassium; Na, sodium; SBP, systolic BP.

### Resting State

As shown in Table 2, no significant differences were observed between groups in HRV and BPV parameters in either the time or frequency domain during the 5-minute rest period. Spontaneous BRS, assessed using both spectral and sequential methods, also did not differ between groups.

**Table 2:** Results of heart rate variability, BP variability, and spontaneous baroreflex sensitivity indexes at rest

Parameters	ADPKD (*n*=17)	Controls (*n*=19)	*P* Value
**Time domain—HRV**			
RR interval, ms	833.2±119.0	819.4±106.8	0.72
SD, ms	65 (43–75)	58 (49–76)	0.92
RMSSD, ms	48 (27–57)	40 (29–54)	0.86
**Frequency domain—HRV**			
VLF abs, ms^2^	663 (330–1642)	563 (368–825)	0.48
LF abs, ms^2^	1205 (663–1876)	1081 (739–2970)	0.62
HF abs, ms^2^	950 (297–1457)	858 (487–1672)	0.89
LF, nu	56.6±14.4	59.7±18.1	0.57
HF, nu	43.4±14.4	40.3±18.1	0.57
LF/HF, au	2.1±1.4	2.6±1.8	0.33
**Time domain—BPV**			
SD, mm Hg	6.28±1.95	6.59±1.80	0.34
**Frequency domain—BPV**			
VLF abs, mm Hg^2^	8.6 (5.0–14.4)	8.1 (5.5–21.0)	0.59
LF abs, mm Hg^2^	11.6 (8.0–18.8)	16.0 (9.8–20.1)	0.56
HF abs, mm Hg^2^	2.6 (1.8–3.9)	2.8 (2.2–4.9)	0.46
**BRS—spectral method**			
BRS, ms/mm Hg	9.9 (7.0–12.2)	10.0 (7.8–12.8)	0.93
**BRS—sequential method**			
BRS, ms/mm Hg	14.6 (8.6–21.0)	10.6 (8.6–14.8)	0.37

Values are means±SD or median (interquartile range). Groups were contrasted *via* the independent samples Student *t* test. Abs, absolute unit; ADPKD, autosomal dominant polycystic kidney disease; au, arbitrary unit; BPV, BP variability; BRS, baroreflex sensitivity; HF, high frequency; HRV, heart rate variability; LF, low frequency; nu, normalized unit; RMSSD, root mean square of successive differences; RR interval, time between two consecutive R waves on an electrocardiogram; SD, SD of RR interval; VLF, very low frequency.

### VM

**Figure 1:**
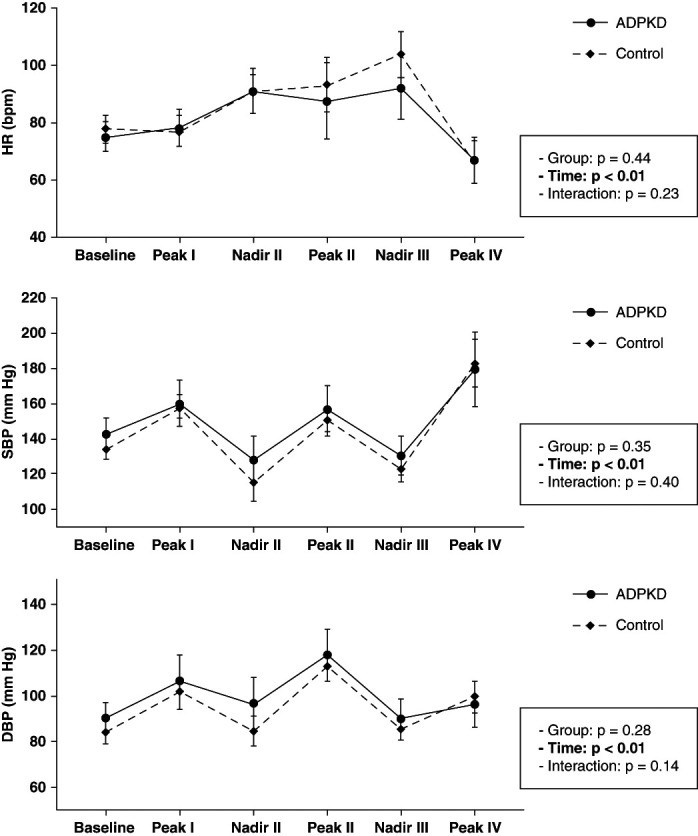
**Cardiovascular response to the VM in patients with ADPKD (solid line) and healthy controls (dashed line).** Graphs show the mean values (95% confidence interval) of HR, SBP, and DBP at baseline and during each phase. Repeated measures ANOVA revealed a significant effect of time for all variables (*P* < 0.01), consistent with the expected physiological autonomic response, with no significant differences between groups (HR: *P* = 0.44; SBP: *P* = 0.35; DBP: *P* = 0.28) and no group-by-time interaction (*P* > 0.14). ADPKD, autosomal dominant polycystic kidney disease; DBP, diastolic BP; HR, heart rate; SBP, systolic BP; VM, Valsalva maneuver.

As shown in Figure 1, the mean values of SBP, DBP, and HR varied across the phases of the VM but did not differ between groups. Similarly, BRS measures—BRSv, BRSa, and global BRS—also showed no significant differences between groups (Table 3).

**Table 3:** Valsalva maneuver indexes

Parameters	ADPKD (*n*=16)	Controls (*n*=18)	*P* Value
PRT, s	0.724 (0.599–0.982)	0.737 (0.553–1.232)	0.77
VR	1.8 (1.4–2.1)	1.8 (1.6–2.6)	0.14
BRSv (ms/mm Hg)	3.2 (2.7–4.7)	2.7 (1.9–4.0)	0.24
BRSa (mm Hg/s)	6.7 (5.7–19.8)	13.8 (9.6–21.6)	0.31
BRSg (ms/s)	34 (22.3–58.4)	25.8 (10.7–65.0)	0.49

Values are median (interquartile range). Groups were contrasted *via* the independent samples Student *t* test. ADPKD, autosomal dominant polycystic kidney disease; BRSa, adrenergic baroreflex sensitivity; BRSg, global baroreflex sensitivity; BRSv, vagal baroreflex sensitivity; PRT, pressure recovery time; VR, Valsalva ratio.

### Sympathoexcitatory Maneuvers

**Figure 2:**
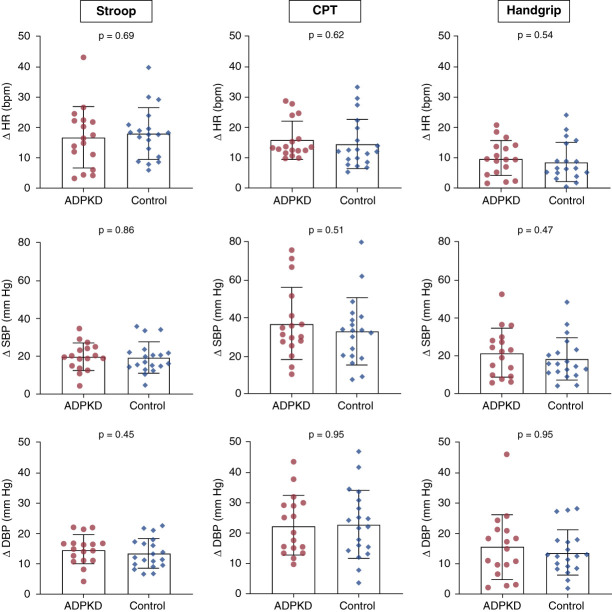
**Cardiovascular reactivity to the Stroop test, handgrip, and CPT in ADPKD (*n*=18) and control (*n*=19) groups.** Groups were contrasted *via* the independent samples Student *t* test. CPT, cold pressor test.

Cardiovascular reactivity to the Stroop, handgrip, and CPT was not different between groups (Figure 2).

### TKV

HtTKV by magnetic resonance imaging was available for 17 patients with ADPKD. The median HtTKV was 256 (235–385) ml/m. According to MIC, eight patients were classified as slow progressors (1A: *n*=4; and 1B: *n*=4) and nine of them as rapid progressors (1C: *n*=6, 1D: *n*=2, 1E: *n*=1). The median HtTKV was 225 ml/m in slow progressors and 382 ml/m in rapid progressors (data not shown in tables). No significant correlation was found between HtTKV and any parameter obtained from the autonomic tests (Supplemental Table 1).

### Autonomic Function Comparison Based on MIC

No differences were observed between rapid and slow progressors in terms of age, BMI, and eGFR (Supplemental Table 2). Similarly, there were no differences in baseline hemodynamics, HRV, BPV, or BRS during rest. HR and SBP responses to the VM also did not differ between the two groups (Supplemental Figure 1). In addition, no significant differences were found in PRT, Valsalva ratio, or BRS (Supplemental Table 2). Regarding the sympathoexcitatory maneuvers, no differences were observed between groups for delta HR, SBP, and DBP (Supplemental Table 3).

### Serum and Urinary Markers

Serum levels of IL-6, TNF-*α*, NPY, ADMA, and AGT did not differ between patients with ADPKD and controls (Table 4). Regarding urinary parameters, uMCP1/uCr showed no difference between groups, whereas uAGT/uCr was significantly higher in the ADPKD group compared with controls (*P* < 0.03). Similarly, albuminuria (uAlb/uCr), although within the normal range, was statistically higher in patients with ADPKD than in controls (*P* < 0.01). A positive correlation was found between uAGT/uCr and uAlb/uCr (*r*=0.763, *P* < 0.001). No significant correlations were observed between uAGT/uCr or uAlb/uCr with any parameters from the autonomic assessments at rest or during sympathoexcitatory maneuvers (Supplemental Table 4). Furthermore, uAGT/uCr and uAlb/uCr did not correlate with HtTKV in patients with ADPKD (Supplemental Table 5).

**Table 4:** Serum and urinary markers

Parameters	ADPKD (*n*=14)	Controls (*n*=11)	*P* Value
**Serum markers**			
IL-6, pg/ml	0.64 (0.43–0.97)	0.43 (0.32–0.86)	0.34
TNF-*α*, pg/ml	0.42 (0.35–0.50)	0.43 (0.39–0.48)	0.68
NPY, pg/ml	1222 (845–2151)	718 (548–1085)	0.25
ADMA, *μ*mol/L	0.49 (0.38–0.50)	0.44 (0.41–0.47)	0.54
sAGT, *μ*g/ml	18.5 (16.0–21.4)	16.5 (12.5–17.8)	0.29
**Urinary markers**			
uAGT/uCr (*μ*g/g)	6.13 (1.20–9.57)	3.92 (2.68–4.33)	0.03
uAlb/uCr (mg/g)	15.2 (10.1–21.1)	4.2 (3.0–5.7)	0.01
uMCP1/uCr (pg/mg)	2.35 (1.64–3.12)	1.68 (1.15–1.96)	0.13

Values are median (interquartile range). Groups were contrasted *via* the independent samples Student *t* test. ADMA, asymmetric dimethylarginine; ADPKD, autosomal dominant polycystic kidney disease; NPY, neuropeptide Y; sAGT, serum angiotensinogen; uAGT, urinary angiotensinogen; uAlb/uCr, albuminuria; uMCP1, urinary monocyte chemoattractant protein-1.

## Discussion

Early-onset hypertension is a critical clinical biomarker associated with ADPKD severity because of its contribution to both cystic burden and kidney function decline.^57^ Significant differences in renal survival have been observed between patients treated for hypertension before age 35 years, highlighting this cardiovascular manifestation as a modifiable risk factor whose treatment may improve outcomes.^58^ However, the mechanisms that trigger hypertension in ADPKD remain unclear.^11^ Therefore, this study aimed to investigate whether cardiovascular autonomic dysregulation occurs in patients with ADPKD before hypertension and renal function decline. Despite a comprehensive assessment of cardiovascular autonomic function at rest and during sympathoexcitatory maneuvers, we found no evidence of cardiovascular autonomic abnormalities in our cohort. These findings suggest that autonomic dysregulation does not play a chief role in triggering hypertension in ADPKD. Instead, other mechanisms, such as intrarenal RAAS activation, could be more relevant in an early stage of the ADPKD natural history.

Some studies in animal models of recessive polycystic kidney disease reported abnormal autonomic cardiovascular regulation at the hypertensive stage of the disease.^59,60^ The single study investigating the time course of cardiovascular autonomic function from normotension to hypertension used the two kidneys-one clip model.^22^ This study found that spontaneous cardiac BRS was reduced before the onset of hypertension, and it was the first autonomic variable to be altered versus a control group among several HRV and BPV indexes. However, it is important to note that the renal hypoperfusion in the two kidneys-one clip model is plausibly greater than in ADPKD, limiting data extrapolation. Therefore, a similar time course analysis is warranted in a nonorthologous ADPKD model. Similar to the studies in animal models, clinical studies that investigated autonomic cardiovascular regulation in patients with ADPKD included only hypertensive patients^29,31^ with the exception of the study by Klein *et al.*,^30^ which included a group of normotensive patients with ADPKD with preserved renal function. The authors found no difference in resting muscle sympathetic nerve activity nor the sympathetic response to pharmacologically induced step changes in BP between these patients and controls. Herein, we sought to advance available evidence in normotensive patients with ADPKD, assessing complementary autonomic indexes at rest and during the VM. More importantly, we examined the cardiovascular reactivity to sympathoexcitatory maneuvers because large cohort studies support that this reactivity can predict hypertension surge in otherwise normotensive healthy individuals. Despite such comprehensive cardiovascular autonomic assessment, we found no difference in any index between patients and controls, indicating that it is unlikely that the autonomic nervous system triggers hypertension development in ADPKD. It may instead contribute to hypertension worsening after its advent.

The kidneys of hypertensive patients with ADPKD have enhanced nerve density in the periadventitial tissue of the renal artery than hypertensive individuals without ADPKD.^61^ Moreover, renal denervation in animal models nonorthologous to human ADPKD reduced the size of cysts,^62^ suggesting that cysts' size depends on renal innervation. Therefore, it is plausible that the higher the TKV, the higher the renal afferent density. Then, excessive renal afferents signaling to brainstem autonomic neurons could impair the autonomic control of the cardiovascular system. Therefore, we further analyzed our data according to MIC to test this hypothesis. However, we found no difference in HRV, BPV, and BRS at rest, HR and SBP responses to the VM, and cardiovascular reactivity to sympathoexcitatory maneuvers between patients with ADPKD who were rapid or slow progressors. Although the distribution of slow and rapid progressors was similar within the ADPKD group, the small number of patients in each classification and the higher representation of rapid progressors in 1C rather than 1D or 1E MIC precludes a definite refusal of a link between TKV and autonomic cardiovascular regulation in normotensive patients with ADPKD. In addition, this issue deserves further investigation in patients with more advanced disease.

Our data demonstrated no differences in markers of inflammation (*i.e*., IL-6 and TNF-*α*), as previously described in some series from initial stages of the disease.^63–68^ Serum level of ADMA, an important inhibitor of nitric oxide synthase, was also not different between ADPKD and controls, in contrast to findings in the literature.^69–71^ Similarly, in a previous study from our group,^72^ basal ADMA levels were not altered in young normotensive patients with ADPKD versus controls. However, ADMA did not decrease after incremental exercise in a cycle ergometer in patients with ADPKD as it did in controls, suggesting the presence of endothelial dysfunction only after an intense physiological stimulus. As we measured inflammatory and endothelial function markers only at rest, we could not verify if a similar phenomenon would happen in the current series.

Urinary MCP-1, an important biomarker of progression in ADPKD,^73,74^ was not elevated in the present sample of patients with ADPKD, probably because of the early stages of the disease. Similarly, serum NPY, a sympathetic neurotransmitter with broad effects on the central nervous system, heart, bones, and kidneys,^75,76^ did not differ between ADPKD and healthy controls in the present series, but such parameter has been reported as increased only in later stages of CKD from other etiologies and not in ADPKD.^77,78^ Of note, uAGT, but not sAGT, was statistically higher in the ADPKD group versus controls, demonstrating greater activity of intrarenal RAAS. Because sAGT is not filtered by the glomeruli because of its molecular size, uAGT reflects only the activity of the intrarenal renin-angiotensin system (RAS). Therefore, uAGT had already been suggested as an effective biomarker of intrarenal RAS overactivity in hypertension.^79^ Studies in patients with ADPKD have disclosed increased uAGT, especially among hypertensive older patients.^80,81^ Interestingly, Salih *et al.*^82^ observed that increased levels of uAGT was more associated with hypertension in later stages of ADPKD when compared with CKD of other etiologies, suggesting that this biomarker is associated with ADPKD itself. Nevertheless, the findings in normotensive patients with ADPKD are still controversial.^17,18,80–83^ Given that the present series consisted of young, normotensive patients with ADPKD with preserved renal function, the higher levels of uAGT support overactivity of intrarenal RAS already in the early stages of the disease, as evidenced in experimental models,^84^ representing a prognostic biomarker of future hypertension in the context of ADPKD. The lack of correlation between uAGT and HtTKV in this series might have been accounted for by the mild increases in median HtTKV in such early stages of ADPKD. It is well established that Ang II increases the permeability of the glomerular filtration barrier for albumin and initiates albumin endocytosis by podocytes.^85^ Pontes *et al.*,^19^ in an experimental study undertaken in our laboratory, observed that acute stimulation of renal sympathetic nervous activity modulates sodium reabsorption, increasing intrarenal generation of Ang II and activation of the angiotensin II type 1 receptor in the luminal membrane, independent of changes in systemic BP, renal blood flow, and glomerular filtration. Furthermore, our group showed reduced proteinuria, albuminuria, and renal sympathetic activity after selective afferent renal denervation in a renovascular model.^23^ Although albuminuria levels were significantly elevated in patients with ADPKD in our sample, they remained within the normal reference range, making their clinical significance unclear. Nevertheless, the potential relevance of this increase—as a marker of intrarenal RAAS overactivity in early ADPKD—cannot be ruled out. The direct correlation between albuminuria and uAGT levels further supports this hypothesis. Therefore, we speculate that renal afferent signaling may be involved in the initial activation of the intrarenal RAAS in ADPKD, which could reflexively enhance renal sympathetic activity and thereby contribute to urinary albumin excretion. Furthermore, it is also possible to speculate that the renal sympathetic activity is not mirrored by the sympathetic control of other territories because we found no evidence of sympathetic overactivation to the heart and systemic vessels in normotensive patients with ADPKD.

This study employed strict inclusion criteria, focusing specifically on young patients with ADPKD without hypertension, obesity, or renal dysfunction, among other factors. Consequently, enrolling a larger number of participants was not feasible. In addition, we assessed autonomic function through cardiovascular responses rather than direct intraneural measurements. Although the current statistical analyses were appropriate, the relatively small sample size might have limited the power to detect small or moderate differences, particularly in physiological and biochemical measures. To address both limitations, we used a comprehensive set of validated autonomic proxies during undisturbed rest and sympathoexcitatory maneuvers, which is a strength of the study design. Therefore, it is unlikely that the lack of statistical significance across all tests and conditions can be attributed solely to the small sample size. Notably, some of these indices are sensitive predictors of incident hypertension, making it unlikely that a different type of cardiovascular autonomic assessment would alter the interpretation of our findings. Third, perceived pain, mental stress, and physical effort are known to influence cardiovascular reactivity during the CPT, stroop, and handgrip, respectively. In our study, both patients and controls reported similar levels of perceived pain and mental stress during the CPT and Stroop. However, patients reported a smaller increase in perceived physical effort during the handgrip compared with controls. Although we cannot provide a clear explanation for this finding, we minimized bias in interpreting cardiovascular reactivity by comparing changes in HR, SBP, and DBP at similar levels of perceived physical effort. Finally, the urine biomarkers smaller sample size might have compromised the comparisons with control group, deserving additional investigation in future studies. Nevertheless, uAGT, the most important of these parameters, was found to be significantly higher despite such limitations.

In summary, this study did not demonstrate cardiovascular autonomic dysregulation in normotensive patients with ADPKD with preserved renal function, nor in those classified as rapid progressors according to the MIC compared with slow progressors. Serum markers of endothelial function and inflammation did not differ between patients and controls or between rapid and slow progressors. However, patients with ADPKD exhibited higher levels of albuminuria and uAGT compared with controls, with a direct correlation observed between these parameters, suggesting activation of the intrarenal RAAS even in the initial stages of the disease. This abnormality may contribute to a premature rise in systemic arterial pressure in patients with ADPKD, although neurohumoral dysregulation likely emerges later as the disease progresses, playing a role in the development and persistence of hypertension.

## Supplementary Material

SUPPLEMENTARY MATERIAL

## Data Availability

Original data generated for the study will be made available upon reasonable request to the corresponding author. Raw Data/Source Data. Data are subject to institutional ethical restrictions and will be provided by the corresponding author upon justified request.

## Declarative Statements

This study includes clinical experimentation and received Institutional Review Board or Ethics Committee approval. All patients provided written informed consent. This study includes clinical experimentation and complies with the Declaration of Helsinki.

## Supplemental Material

Supplemental Table 1. Pearson correlation coefficients between HtTKV and indexes of autonomic test.

Supplemental Figure 1. Variations in HR, SBP, and DBP during the VM according to MIC.

Supplemental Table 2. Results of HRV, BPV, and spontaneous BRS indexes during rest period divided for MIC.

Supplemental Table 3. Autonomic control assessment indexes in the VM divided for MIC.

Supplemental Table 4. Pearson correlation coefficients between urinary AGT/Cr and urinary Alb/Cr with the indexes of autonomic tests at rest and VM.

Supplemental Table 5. Pearson correlation coefficients between HtTKV and inflammatory, hormonal, and endothelial function markers.
