# Supplementary material for: Cardiovascular Autonomic Control in Normotensive Patients with Autosomal Dominant Polycystic Kidney Disease
Source: Kidney360. 2025 Aug 28;6(12):2185–95. doi: 10.34067/KID.0000000958 (PMC12708371; doi:10.34067/KID.0000000958)
Supplement: SUPPLEMENTARY MATERIAL [file kidney360-6-2185-s001.pdf]

ASN
American Society of Nephrology
JASN
Journal of the American Society of Nephrology
KIDNEY360
Accessing Our World From Every Angle
CJASN
Clinical Journal of the American Society of Nephrology

ASN Journal Disclosure Form

As per ASN journal policy, I have disclosed any financial relationships or commitments I have held in the past 36 months as included below. I have listed my Current Employer below to indicate there is a relationship requiring disclosure. If no relationship exists, my Current Employer is not listed.

A. Anauate reports the following:
Employer: Universidade Federal de São Paulo

I understand that the information above will be published within the journal article, if accepted, and that failure to comply and/or to accurately and completely report the potential financial conflicts of interest could lead to the following: 1) Prior to publication, article rejection, or 2) Post-publication, sanctions ranging from, but not limited to, issuing a correction, reporting the inaccurate information to the authors' institution, banning authors from submitting work to ASN journals for varying lengths of time, and/or retraction of the published work.

Name: Ana Carolina Anauate
Manuscript ID: K360-2025-000437R1
Manuscript Title: Cardiovascular autonomic control in normotensive patients with Autosomal Dominant Polycystic Kidney Disease
Date of Completion: July 14, 2025
Disclosure Updated Date: July 14, 2025

ASN Journal Disclosure Form

As per ASN journal policy, I have disclosed any financial relationships or commitments I have held in the past 36 months as included below. I have listed my Current Employer below to indicate there is a relationship requiring disclosure. If no relationship exists, my Current Employer is not listed.

C. Bergamaschi has nothing to disclose.

I understand that the information above will be published within the journal article, if accepted, and that failure to comply and/or to accurately and completely report the potential financial conflicts of interest could lead to the following: 1) Prior to publication, article rejection, or 2) Post-publication, sanctions ranging from, but not limited to, issuing a correction, reporting the inaccurate information to the authors' institution, banning authors from submitting work to ASN journals for varying lengths of time, and/or retraction of the published work.

Name: Cassia T. Bergamaschi
Manuscript ID: ID: K360-2025-000437R1
Manuscript Title: Cardiovascular autonomic control in normotensive patients with Autosomal Dominant Polycystic Kidney Disease
Date of Completion: July 15, 2025
Disclosure Updated Date: July 15, 2025

ASN American Society of Nephrology
JASN Journal of the American Society of Nephrology
KIDNEY360 Accessing Our World From Every Angle
CJASN Clinical Journal of the American Society of Nephrology

ASN Journal Disclosure Form

As per ASN journal policy, I have disclosed any financial relationships or commitments I have held in the past 36 months as included below. I have listed my Current Employer below to indicate there is a relationship requiring disclosure. If no relationship exists, my Current Employer is not listed.

R. Campos reports the following:
Employer: Federal University of São Paulo - School fo Medicine

I understand that the information above will be published within the journal article, if accepted, and that failure to comply and/or to accurately and completely report the potential financial conflicts of interest could lead to the following: 1) Prior to publication, article rejection, or 2) Post-publication, sanctions ranging from, but not limited to, issuing a correction, reporting the inaccurate information to the authors' institution, banning authors from submitting work to ASN journals for varying lengths of time, and/or retraction of the published work.

Name: Ruy R. Campos
Manuscript ID: K360-2025-000437R1
Manuscript Title: Cardiovascular autonomic control in normotensive patients with Autosomal Dominant Polycystic Kidney Disease
Date of Completion: July 16, 2025
Disclosure Updated Date: July 16, 2025

ASN American Society of Nephrology JASN Journal of the American Society of Nephrology KIDNEY360 Accessing Our World From Every Angle CJASN Clinical Journal of the American Society of Nephrology

ASN Journal Disclosure Form

As per ASN journal policy, I have disclosed any financial relationships or commitments I have held in the past 36 months as included below. I have listed my Current Employer below to indicate there is a relationship requiring disclosure. If no relationship exists, my Current Employer is not listed.

I. Heilberg has nothing to disclose.

I understand that the information above will be published within the journal article, if accepted, and that failure to comply and/or to accurately and completely report the potential financial conflicts of interest could lead to the following: 1) Prior to publication, article rejection, or 2) Post-publication, sanctions ranging from, but not limited to, issuing a correction, reporting the inaccurate information to the authors' institution, banning authors from submitting work to ASN journals for varying lengths of time, and/or retraction of the published work.

Name: Ita Pfeferman Heilberg
Manuscript ID: K360-2025-000437R1
Manuscript Title: Cardiovascular autonomic control in normotensive patients with Autosomal Dominant Polycystic Kidney Disease
Date of Completion: July 14, 2025
Disclosure Updated Date: July 14, 2025

ASN
American Society of Nephrology
JASN
Journal of the American Society of Nephrology
KIDNEY360
Accessing Our World From Every Angle
CJASN
Clinical Journal of the American Society of Nephrology

ASN Journal Disclosure Form

As per ASN journal policy, I have disclosed any financial relationships or commitments I have held in the past 36 months as included below. I have listed my Current Employer below to indicate there is a relationship requiring disclosure. If no relationship exists, my Current Employer is not listed.

M. Ormanji reports the following:
Employer: Universidade Federal de Sao Paulo

I understand that the information above will be published within the journal article, if accepted, and that failure to comply and/or to accurately and completely report the potential financial conflicts of interest could lead to the following: 1) Prior to publication, article rejection, or 2) Post-publication, sanctions ranging from, but not limited to, issuing a correction, reporting the inaccurate information to the authors' institution, banning authors from submitting work to ASN journals for varying lengths of time, and/or retraction of the published work.

Name: Milene Subtil Ormanji
Manuscript ID: K360-2025-000437R1
Manuscript Title: Cardiovascular autonomic control in normotensive patients with Autosomal Dominant Polycystic Kidney Disease
Date of Completion: July 15, 2025
Disclosure Updated Date: July 15, 2025

ASN American Society of Nephrology · JASN Journal of the American Society of Nephrology · KIDNEY360 Accessing Our World From Every Angle · CJASN Clinical Journal of the American Society of Nephrology

ASN Journal Disclosure Form

As per ASN journal policy, I have disclosed any financial relationships or commitments I have held in the past 36 months as included below. I have listed my Current Employer below to indicate there is a relationship requiring disclosure. If no relationship exists, my Current Employer is not listed.

D. Rocha reports the following:
Employer: Universidade Federal de São Paulo (UNIFESP)

I understand that the information above will be published within the journal article, if accepted, and that failure to comply and/or to accurately and completely report the potential financial conflicts of interest could lead to the following: 1) Prior to publication, article rejection, or 2) Post-publication, sanctions ranging from, but not limited to, issuing a correction, reporting the inaccurate information to the authors' institution, banning authors from submitting work to ASN journals for varying lengths of time, and/or retraction of the published work.

Name: Daniel Ribeiro da Rocha
Manuscript ID: K360-2025-000437R1
Manuscript Title: Cardiovascular autonomic control in normotensive patients with Autosomal Dominant Polycystic Kidney Disease
Date of Completion: July 15, 2025
Disclosure Updated Date: July 15, 2025

ASN American Society of Nephrology | JASN Journal of the American Society of Nephrology | KIDNEY360 Accessing Our World From Every Angle | CJASN Clinical Journal of the American Society of Nephrology

ASN Journal Disclosure Form

As per ASN journal policy, I have disclosed any financial relationships or commitments I have held in the past 36 months as included below. I have listed my Current Employer below to indicate there is a relationship requiring disclosure. If no relationship exists, my Current Employer is not listed.

B. Silva reports the following:
Employer: Universidade Federal de São Paulo

I understand that the information above will be published within the journal article, if accepted, and that failure to comply and/or to accurately and completely report the potential financial conflicts of interest could lead to the following: 1) Prior to publication, article rejection, or 2) Post-publication, sanctions ranging from, but not limited to, issuing a correction, reporting the inaccurate information to the authors' institution, banning authors from submitting work to ASN journals for varying lengths of time, and/or retraction of the published work.

Name: Bruno Moreira Silva
Manuscript ID: K360-2025-000437
Manuscript Title: Cardiovascular autonomic control in normotensive patients with Autosomal Dominant Polycystic Kidney Disease
Date of Completion: July 15, 2025
Disclosure Updated Date: July 15, 2025
