# Supplementary material for: Cardiovascular Autonomic Control in Normotensive Patients with Autosomal Dominant Polycystic Kidney Disease
Source: Kidney360. 2025 Aug 28;6(12):2185–95. doi: 10.34067/KID.0000000958 (PMC12708371; doi:10.34067/KID.0000000958)
Supplement: SUPPLEMENTARY MATERIAL [file kidney360-6-2185-s002.pdf]

Supplemental Data

Supplemental Table 1: Pearson correlation coefficients between HtTKV and indexes of autonomic test

Parameters		Log_HtTKV
**RRI (ms)**	Pearson's R	0,465
	p-value	0,081
**SD (ms)**	Pearson's R	0,420
	p-value	0,119
**RMSSD (ms)**	Pearson's R	0,345
	p-value	0,209
**LF (nu)**	Pearson's R	-0,159
	p-value	0,571
**HF (nu)**	Pearson's R	0,159
	p-value	0,571
**LF/HF**	Pearson's R	0,017
	p-value	0,953
**PRT (s)**	Pearson's R	-0,156
	p-value	0,579
**VR**	Pearson's R	-0,019
	p-value	0,946
**BRSv**	Pearson's R	0,076
	p-value	0,787
**BRSa**	Pearson's R	-0,132
	p-value	0,639
**BRSg**	Pearson's R	0,123
	p-value	0,661
**ΔSBP_CPT**	Pearson's R	-0,038
	p-value	0,889
**ΔSBP_HG**	Pearson's R	0,356
	p-value	0,176
**ΔSBP_Stroop**	Pearson's R	0,054
	p-value	0,843
**ΔDBP_CPT**	Pearson's R	0,038
	p-value	0,889
**ΔDBP_HG**	Pearson's R	0,406
	p-value	0,119
**ΔDBP_Stroop**	Pearson's R	0,030
	p-value	0,913
**ΔHR_CPT**	Pearson's R	-0,036
	p-value	0,894
**ΔHR_HG**	Pearson's R	0,084
	p-value	0,756
**ΔHR_Stroop**	Pearson's R	-0,219
	p-value	0,416

HtTKV: total renal volume adjusted for height. RRI: RR interval. SD: SD of all normal RRI. RMSSD: root mean square of the square of the differences between adjacent normal RRI. LF: low frequency. HF: high frequency. abs: absolute. ms: milliseconds. S: seconds. PRT: blood pressure recovery time in phase III of VM, VR: Valsalva ratio, BRSv, BRSa and BRSg: Vagal, Adrenergic and Global Baroreflex Sensitivity, respectively. HR: heart rate; SBP and DBP: systolic and diastolic blood pressure.

Supplemental Data

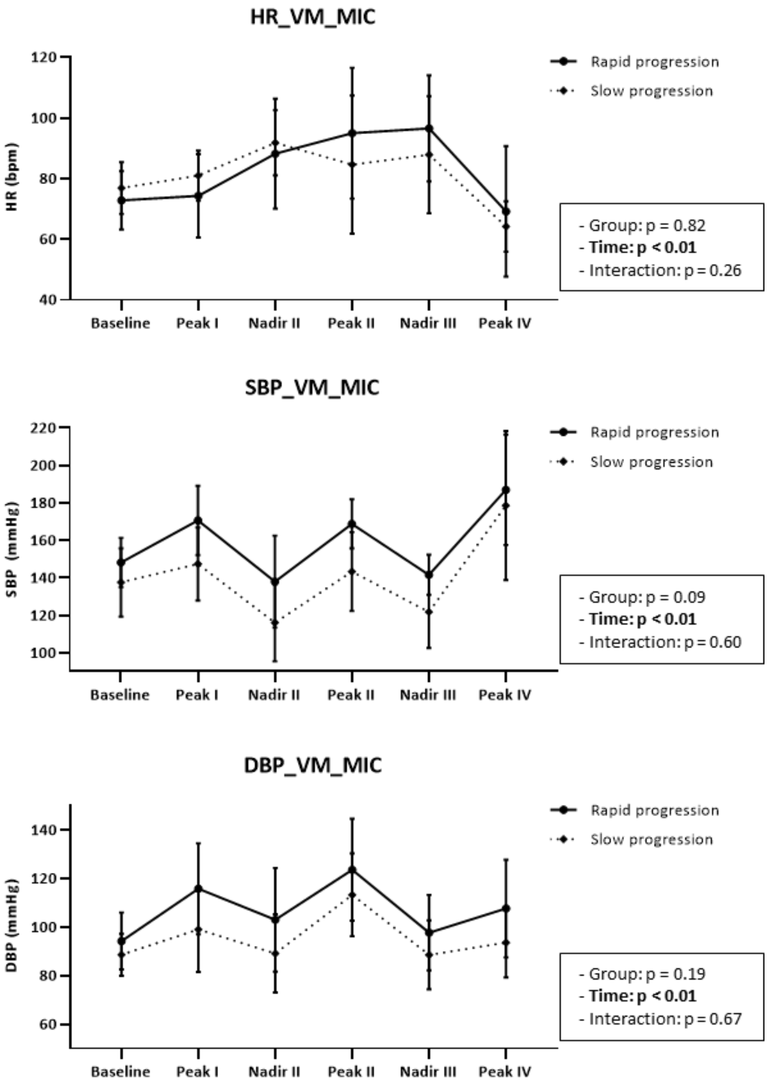

Supplemental Figure 1 - Variations in HR, SBP, and DBP during the Valsalva maneuver according to Mayo Imaging Classification (MIC). I to IV correspond to the phases of the VM. N = 8 for both groups. HR: heart rate. SBP and DBP: systolic and diastolic blood pressure. Groups contrasted using two-way ANOVA with repeated measures.

Supplemental Data

Supplemental Table 2 - Results of HRV, BPV and spontaneous BRS indexes during rest period divided for Mayo Imaging Classification

	Rapid progression (N=8)	Slow progression (N=8)	p value
**Time domain - HVR**			
RRI, ms	858.1 ± 148.9	811.3 ± 94.3	0.46
SDNN, ms	73 ± 31	55 ± 21	0.19
RMSSD, ms	50 ± 25	40 ± 25	0.44
**Frequency domain - HRV**			
VLF abs, ms^2	1249 (580-2344)	468 (252-661)	0.14
LF abs, ms^2	1412 (1054-2005)	563 (435-1782)	0.10
HF abs, ms^2	1159 (369-1702)	469 (273-1110)	0.32
LF, nu	60 ± 18	55 ± 10	0.51
HF, nu	40 ± 18	44 ± 10	0.51
LF/HF	2.7 ± 1.8	1.7 ± 0.7	0.17
**Time domain - BPV**			
SD, mmHg	6.7 ± 1.9	5.9 ± 2.2	0.34
**Frequency domain - BPV**			
VLF abs, $mmHg^2$	13.8 (8.0-14.7)	6.1 (4.4-14.0)	0.11
LF abs, $mmHg^2$	16.3 (8.1-27.8)	9.9 (6.9-12.3)	0.28
HF abs, $mmHg^2$	2.7 (1.7-3.7)	2.3 (1.6-4.4)	0.81
**Baroreflex sensitivity - Spectral method**			
BRS, ms/mmHg	10.1 (7.3-14.1)	8.5 (6.6-11.2)	0.44
**Baroreflex sensitivity - Sequential method**			
BRS, ms/mmHg	16.7 (8.3-21.4)	10.0 (7.7-21.4)	0.67

Values are means ± SD or median (interquartile range). ADPKD, Autosomal Dominant Polycystic Kidney Disease; HRV and BPV, Heart rate and blood pressure variability; BRS, Baroreflex sensitivity; RRI, RR interval; SDNN, SD of all normal RRI; RMSSD, Root mean square of the square of the differences between adjacent normal RRI; VLF, Very low frequency; LF, Low frequency; HF, High frequency; Abs, Absolute; Nu, Normalized units. Groups were contrasted via the independent samples Student's t test. Missing: 1 ADPKD group (ECG and BP tracing unable to be assessed).

Supplemental Data

Supplemental Table 3 - Autonomic control assessment indexes in the Valsalva Maneuver divided for Mayo Imaging Classification

	Rapid progression (N=7)	Slow progression (N=8)	p value
PRT, s	0.685 (0.583 - 0.732)	0.930 (0.649 - 1.029)	0.39
VR	2.1 (1.7 - 2.3)	1.7 (1.4 - 1.8)	0.38
BRSv (ms/mmHg)	3.3 (2.9 - 6.7)	3.4 (2.3 - 4.0)	0.39
BRSa (mmHg/s)	6 (5.0 - 10.3)	16.9 (6.1 - 20.7)	0.18
BRSg (ms/s)	28.9 (16.5 - 53.8)	42.1 (28.4 - 66.4)	0.56

Values are median (interquartile range). ADPKD, Autosomal Dominant Polycystic Kidney Disease; PRT, pressure recovery time; VR, Valsalva ratio; BRS, Baroreflex sensitivity; v, vagal; a, adrenergic; g, global. Groups were contrasted via the independent samples Student’s t test. Missing: 1 ADPKD group (ECG and BP tracing unable to be assessed).

Supplemental Data

Supplemental Table 4 - Pearson correlation coefficients between urinary AGT/Cr and urinary Alb/Cr with the indexes of autonomic tests at Rest and Valsalva maneuver

Parameters		Log_uAlb/uCr	Log_uAGT/uCr
**Rest HR**	Pearson's R	0,332	0,287
	p-value	0,112	0,184
**Rest SBP**	Pearson's R	0,146	0,001
	p-value	0,496	0,997
**Rest DBP**	Pearson's R	0,344	0,216
	p-value	0,100	0,323
**RRI (ms)**	Pearson's R	-0,306	-0,195
	p-value	0,146	0,372
**SD (ms)**	Pearson's R	-0,172	-0,039
	p-value	0,420	0,860
**RMSSD (ms)**	Pearson's R	-0,168	0,051
	p-value	0,432	0,816
**LF (nu)**	Pearson's R	0,078	-0,142
	p-value	0,718	0,519
**HF (nu)**	Pearson's R	-0,078	0,142
	p-value	0,718	0,519
**LF/HF**	Pearson's R	0,174	-0,046
	p-value	0,417	0,834
**PRT (s)**	Pearson's R	0,083	0,011
	p-value	0,727	0,965
**VR**	Pearson's R	-0,269	-0,129
	p-value	0,226	0,578
**BRSv**	Pearson's R	0,269	0,206
	p-value	0,226	0,371
**BRSa**	Pearson's R	-0,155	-0,057
	p-value	0,513	0,817
**BRSg**	Pearson's R	0,108	0,008
	p-value	0,651	0,973

AGT: angiotensinogen. Alb: Albuminuria. Cr: Creatinine. RRI: RR interval. SD: SD of all normal RRI. RMSSD: root mean square of the square of the differences between adjacent normal RRI. LF: low frequency. HF: high frequency. abs: absolute. ms: milliseconds. S: seconds. PRT: blood pressure recovery time in phase III of VM, VR: Valsalva ratio, BRSv, BRSa and BRSg: Vagal, Adrenergic and Global Baroreflex Sensitivity, respectively. HR: heart rate; SBP and DBP: systolic and diastolic blood pressure.

Supplemental Data

Supplemental Table 5 - Pearson correlation coefficients between HtTKV and inflammatory, hormonal and endothelial function markers

Parameters		Log_HtTKV
Log_uAlb/Cr	Pearson's R	0,321
	p-value	0,309
Log_NPY	Pearson's R	0,066
	p-value	0,857
Log_IL-6	Pearson's R	0,228
	p-value	0,476
Log_sAGT	Pearson's R	0,066
	p-value	0,839
Log_uAGT	Pearson's R	0,101
	p-value	0,755
Log_TNF-α	Pearson's R	0,229
	p-value	0,474
Log_MCP1	Pearson's R	0,538
	p-value	0,088
Log_ADMA	Pearson's R	0,132
	p-value	0,699

HtTKV: total renal volume adjusted for height. IL-6, Interleukin 6; TNF-α, Tumor necrosis factor alpha; NPY, Neuropeptide Y; ADMA, Asymmetric dimethylarginine; sAGT and uAGT, Serum and urinary angiotensinogen, respectively; uMCP1, Urinary monocyte chemoattractant protein-1. Groups were contrasted via the independent samples Student's t test.
